# Medical Legislation

**Published:** 1845-11-01

**Authors:** 


### EDOITRIAL, MEDICAL INTELLIGENCE, &c.

*Medical Legislation.*—The subject of Medical Legislation has occupied not an inconsiderable degree of attention both in and out of the Profession, in this country and abroad, for several years past. It is a subject which must necessarily continue to occupy attention, until the profession and the public unite in some plan, which will, in the opinion of either, best secure the interests of both. It is not probable that this desirable object will be very soon effected. Should a National Convention assemble agreeably to the recommendation of the New York, State, Medical Society, this will doubtless constitute a prominent topic for the deliberations of that body. It is highly important that members of the profession give to this subject a degree of reflection proportional to its merits, so that they may be qualified to form opinions, and to decide for themselves as to the propriety or practicability of the policy which may be submitted for their approval. We purpose to give, briefly, some of our own views, hoping, at least, that they may serve to suggest in the minds of our readers trains of thought which may lead to satisfactory conclusions.

If the question with the profession were simply this—viz: what is legislation capable of doing for medicine and the medical profession; we should perhaps be disposed to say it could do every thing that is to be desired. We do not profess to have formed an unchangeable opinion on the subject, but we incline to the belief, that if legislation were disposed to exert all its potency, in good faith, in behalf of the best interests of the profession and the public, medicine would thereby derive an impulse which would place it in a position adequate to its full capacities for honor and usefulness. If this question were at all pertinent to the present condition of things, we might stop to consider what course legislation ought to pursue to fulfil to the utmost such a disposition as we have just supposed.

But this is not a question at present before the profession. There is not the shadow of a probability that legislation will place itself in this attitude toward us. The questions which really are for us to consider, are these: What is to be expected from legislation in the existing state of things; and will it be advisable to avail ourselves of what legislation consents to vouchsafe to us? These are questions of a different import, and we are certainly not prepared unhesitatingly to answer the latter in the affirmative.

It is easy for a medical man who has given any reflection to the subject, to perceive what are the requisites on the part of legislation in order for it to discharge its whole duty to the profession and the public. Medical education should be amply provided for; posts of honor and responsibility impartially filled, and rendered accessible to merit alone; the just rights of the profession protected; the qualifications for fellowship raised sufficiently high, and steadily maintained; quackery in every form and shape prohibited by severe penalties—it is needless to multiply similar conditions. Is there the least ground for the expectation that a moiety of these requisites will be advocated or recognized in the halls of legislation? We do not suppose that any member of the profession can be found, whose ardour of enthusiasm is sufficient to lead him to entertain for a moment, any such expectations.

What, then, may be reasonably anticipated, and sought for at the hands of legislation? We cannot reply to this inquiry in detail, but we are free to say, that, in our view, nothing is to be expected from this source which will tend to elevate the profession, and render it either more desirable to its members, or more useful to the public. We incline to the opinion that its present connection with legislation is detrimental both to its members and the public, and that the interests of both would be promoted by an entire disseverance, and an independent organization. Legislation, in our country, is the exponent of public sentiment; and to form an opinion concerning what may be expected from the former, we have only to consider the latter. Even if this connection between them did not exist, it would be utterly impossible to carry out legislative enactments in behalf of our profession so as to render them of any avail, unless they were sustained by public sentiment. Now, it is not to be doubted that public opinion is decidedly adverse to affording any protection, or granting any exclusive privileges to the medical profession. Too many, and too active interests are involved in empirical schemes, and too strong popular prejudices have been excited on the subject, to leave any room for hoping that legislation could be influenced to adopt a stringent protective policy. Indeed, should the profession unite in an endeavor to procure the adoption of this policy, a degree of popular opposition would be at once aroused, which would not only counteract any influence which might be brought to bear upon the object, but would tend to depreciate public estimation of our profession even below the degree in which it is at present held. Even if it were possible to succeed in so far as legislation is concerned, it would be in vain to expect that the policy could be effectually carried out in spite of the resistance which would be offered on every side.

We imagine, then, that the whole subject is narrowed down to this inquiry: Does legislation at this moment confer any benefits upon the Profession? We answer negatively. On the contrary, we are disposed to think, as we have already said, that it is the source of evil rather than of good. What does it actually confer? In most of the States of the Union there are now no laws restricting the practice of medicine, or imposing any disabilities or irregular practitioners. In those where restrictive laws do exist, they are almost entirely nugatory, and may be considered, virtually, repealed. All that legislation does, then, any where, in fact, to establish a distinction between regular and irregular practitioners, is to incorporate Medical Colleges, and thus regulate the distribution of medical degrees. Now does the exercise of this function tend to any advantage? We say it does not, but, on the other hand, that the medical profession would be better without, than with it. We have not space to discuss this question as its merits require, but we suggest it for the consideration of our readers. Our own reflections lead us to believe that if medical schools possessed no legal qualifications whatever to confer degrees—in fact, if legal provisions for diplomas were entirely abandoned, it would be much better for the profession and the public. Our reasons, very briefly, are these: A diploma really has no intrinsic virtue; it is no criterion of medical acquirement or capacity. We deem this sufficiently evident.—The public to some extent, however, think otherwise. In so far as they entertain such notions, we are accessory to an imposition upon their credulity. This is one reason. Another is, we are obliged to recognize, endorse and associate, as matters of course, with any one who has a diploma, although we may be satisfied that his connection with the profession is contributing to bring it into contempt. A third is, that in proportion to the efficacy which a diploma, as such, has with the public, the object of some schools will be, not to afford sound instruction, but to fulfil the student's purpose, viz: to get his degree, for a pecuniary consideration.—Take away the factitious inducement, which, in this respect, Colleges are now able to hold out to pupils whose only desire is to become regularly qualified to practice medicine, and the result would be that they would be compelled to rely upon their real advantages in order to attain to success.

In conclusion, agreeably to the general views which we have expressed in the foregoing remarks, we advocate, at the present time, an entire reliance on a judicious organization, to be wholly independent of all legislation. Let the profession assume the entire responsibility of providing for its character, its elevation, its usefulness and its interests. With regard to the means by which it may hope to secure these objects, we will speak in a future number. We will only say here that we believe self-reliance, with a general and hearty cooperation among its members, will accomplish for it, much, if not most of that improvement which is to be desired. At all events, to this source alone can we look for advancement under existing circumstances. Whether this divorce from legislation will be mutually desired as a permanent arrangement, remains to be seen.—We are by no means positive that a reunion may uot at some future period be desirable; but we are clearly of opinion, that with the present relations and sentiments existing between the profession and the public, it is not to be advocated. If in the progress of events it should appear to the public, as well as to the profession, that their mutual interests would be promoted by legislative protection, it may be adopted with a far better prospect of its being satisfactory to both.

